# Dynamics of the immediate behavioral response to partial social exclusion

**DOI:** 10.1038/s41598-020-80039-0

**Published:** 2021-01-20

**Authors:** J. F. Dewald-Kaufmann, T. Wüstenberg, B. B. Barton, S. Goerigk, M. A. Reinhard, R. Musil, J. Werle, P. Falkai, A. Jobst, F. Padberg

**Affiliations:** 1 Department of Psychiatry and Psychotherapy, University Hospital, Ludwig-Maximilians-University, Munich, Germany; 2 Hochschule Fresenius, University of Applied Sciences, Munich, Germany; 3 Department of Psychiatry and Psychotherapy, Charité Campus Mitte, Charité—Universitätsmedizin Berlin, Berlin, Germany; 4 Department of Clinical Psychology and Psychotherapy, Ruprecht-Karls-University Heidelberg, Heidelberg, Germany; 5 Department of Psychological Methodology and Assessment, Ludwig-Maximilians-University, Munich, Germany

**Keywords:** Psychology, Human behaviour

## Abstract

Social rejection and exclusion (ostracism) represent main stressors in daily life and even threaten mental and physical health. Abundant data from subjective measures in social exclusion paradigms are available, but the dynamic behavioral response is largely unexplored. Here, we applied modified variants of the Cyberball paradigm in two consecutive experiments to investigate the adaptive behavioral and emotional reactions to partial social exclusion. In experiment 1, 68 healthy participants (females, mean age 24.76 ± 4.05 years) played 2 min inclusion, 5 min partial exclusion and 2 min total exclusion. In experiment 2, 94 healthy participants (48 females, mean age 34.50 ± 12.08 years) underwent an experimental condition (2 min inclusion, 10 min partial exclusion) and a control condition (12 min inclusion only) in randomized order. In experiment 1, behavioral responses to partial exclusion showed two characteristics: (1) an immediate increase in ball passes to the excluding player followed (2) by a later return of participants’ behavior to baseline. This finding was replicated for both genders and in comparison to a control condition in experiment 2. The dynamic behavioral response observed here may point to overlapping principles of cooperation in this ball tossing paradigm and serves as a novel experimental proxy.

## Introduction

Social interactions impact individuals’ emotional well-being and social functioning, starting as early as during infancy. Different theories and ideas have been proposed to explain the emotional and behavioral effects of social exclusion, a concept that refers to a social situation in which an individual is kept apart from a group of other individuals^1^. Smart Richman and Leary^2^ capture the diversity of responses to social exclusion by proposing a multi-motive model, that includes different types of reactions: prosocial behavior, withdrawal/avoidance and aggressive/antisocial behavior. Based on their theory, the choice for these reactions is motivated by an interplay between six different behavioral motivations. They state that somebody’s reaction depends on whether (1) he perceives the costs that are caused by the exclusion situation as high, (2) he has the possibility of alternative relationships, (3) he expects that the relationship can be repaired, (4) the relationship is of high value for him, (5) the exclusion situation is chronic and (6) the exclusion is perceived as unfair^2^.

In an alternative model, Williams’ Need Threat Model^3^, the reaction to social exclusion is divided into three stages: according to this model, “social pain” is caused during an early, reflexive stage, in which individuals’ fundamental social needs (belonging, self-esteem, meaningful existence and control) are threatened by the exclusion situation. This reflexive stage is followed by a reflective stage, in which (more rational) coping strategies are applied. Such strategies can for instance be seen in increased motivation to restore threatened fundamental needs by striving towards reconnection with the excluding individual^3–5^. The third phase in this model is the resignation stage, which occurs when social exclusion is experienced over a longer time period, causing feelings of helplessness, alienation, depression and unworthiness^5^. The underlying idea of the reflective stage can also be found in the belongingness hypothesis^6^ or reconnection hypothesis^7,8^. Both state that healthy individuals show a natural tendency to strive for interpersonal relationships and social connections with others, especially in situations in which they feel socially excluded. Similarly, the Social Baseline Theory (SBT)^9^ assumes that human beings seek social proximity in order to regulate their emotions and emotional well-being. According to the SBT, the behavioral reaction to social exclusion can be seen as an automatic, implicit baseline strategy that does not necessarily involve overt decisions or behavior.

The presented theories target different aspects of reactions to social exclusion (e.g., phases of behavioral responses or underlying motives of a specific behavior etc.), all focusing mainly on the individual’s needs, with most of them assuming a prosocial response, reflecting an attempt of reconnection and consequently re-inclusion^7^. However, if this goal has not been achieved, an increase in aggressive impulses and/or feelings^8,10–12^ or emotional numbness^8^ may result in a decrease in prosocial behavior. This observed diversity in reactions following social exclusion supports the multi-motive model by Smart Richman and Leary^2^.

Experimental research that aims to study the effects of social exclusion usually applies paradigms in which participants are negatively evaluated or excluded from a social situation. One experimental paradigm that is widely used is the Cyberball paradigm, a virtual ball game, in which participants receive equal ball tosses from the other players in the beginning but are then socially excluded as they no longer receive balls after a few minutes^4^. The original version by Williams consists of three players, however, versions including more players have also been developed^13^. Observable variables that are affected by the Cyberball paradigm include emotional responses (for a review see ^14,15^) behavioral attention such as aggressive responses^15^ and average ball tosses^16–18^ . Many studies show that experimentally induced social exclusion negatively affects emotional well-being^19,20^. However, Blackhart et al.^14^ concluded in their review that the effects of social exclusion eliminate positive emotions but do not necessarily increase negative emotions. Additionally, Gerber and Wheeler^15^ demonstrated in their meta-analysis that individuals’ needs are threatened by social exclusion and that they therefore strive towards behavioral responses that may be helpful to restore those needs—even if these responses include antisocial behavior, which the authors interpret as an attempt to prioritize the restoration of control rather than belongingness.

With regard to the stages outlined by Williams’, the results of the majority of experiments on social exclusion carried out so far mainly contain information on the reflective stage (rather than the reflexive stage), as emotions were assessed after social exclusion has occurred. Thus, in most experimental studies the immediate effect of social exclusion during the actual situation remains unknown. A meta-analysis aimed to also capture the effect during the reflexive stage and reported a large effect size of the immediate response for the effect of social exclusion on fundamental needs^19^. This effect decreases over time, but ultimately still has a medium strength. However, the authors state that it remains unknown whether their assessment of the immediate response was also influenced by time (and consequently coping strategies), as it was operationalized by asking participants *after* (rather than during) the social exclusion paradigm how they felt *during* the manipulation^19^. Furthermore, the included studies do not study participants’ actual behavior, which may differ from their (retrospective) reports on their emotional responses. Although this approach may represent the most commonly used design to assess the effects of social exclusion, more objective parameters (e.g., neurocognitive correlates^21^; psychophysiological reactions^22^; endocrinological effects^23^) have been studied and demonstrate that the effects are also present on a biological level that may reflect a more immediate response to social exclusion. In addition, the Cyberball paradigm may be seen in a more complex sense than as an experimental tool to measure the effects of social exclusion on an individual level: it can be presumed that besides acting as individuals aiming to fulfill their individual needs, the players also represent a group, which automatically strives to meet and regulate specific social group norms, such as equality between group members (“equality norm”). Following this idea, participants may aim to share balls equally between group members. Once social exclusion occurs, this rule is violated and the other group member may respond with behavioral reactions that aim to represent or even repair those social group norms. This idea has been supported by previous Cyberball studies showing that the observation of social exclusion causes distress^24^ and that participants tend to compensate the effects on group members that are excluded undeservedly^25^. Additionally, Rudert and Greifenender^26^ demonstrated that the effects of social exclusion during a Cyberball game can be affected by the social norm that is attributed to being socially excluded. Similarly, it was shown that the context of the exclusion affected brain activity with higher activation in the right ventral prefrontal cortex in a situation that could not explain social exclusion when compared to a situation in which social exclusion was explained with technical problems^21^, strengthening the idea that social cognitions play an important role when looking at the effects of social exclusion.

As mentioned above, the original Cyberball paradigm includes a group of three players. This is worth mentioning as participants for example tend to play the ball towards the player who did not pass the ball in order to meet certain group norms (i.e. a “no return” rule). In a setting with three players this would imply to pass the ball towards the next player, rather than towards the player who passed it, resulting in a “circular” or “non-circular” playing behavior. Based on this assumption social exclusion would automatically lead to more ball tosses towards the excluding player as participants would receive more ball tosses from the including player and pass them further, here to the excluding, player. In order to interpret behavioral responses to social exclusion, it is important to address this aspect in experimental studies using the Cyberball paradigm.

So far, there is little empirical data on the nature and dynamics of the direct behavioral responses of healthy individuals in situations of social exclusion. Only few Cyberball studies included behavioral reactions during social exclusion, operationalized by ball tosses towards the other players up to this point^16–18^. However, to the best of our knowledge no study has investigated the dynamic change of these direct behavioral responses by looking at ball throws over time. It remains open how healthy individuals immediately react to social exclusion and whether responses include different phases, meaning whether they change over time.

With a modified Cyberball paradigm, enabling to collect data on the direct behavioral responses (here ball tosses) by using a partial exclusion situation, we aim to answer these questions by (1) providing a model of people’s immediate behavioral reactions to social exclusion, and (2) gaining more insight into phase-specific responses, referring to behavioral changes over time.

## Methods

### Procedure

The study comprised two experiments: (1) in a first exploratory experiment, a gender homogeneous sample of women was included. This approach is based on the expected higher effect size and thus statistical power in a female sample. (2) To replicate and generalize possible findings from our first experiment, we conducted a second experiment using a larger, gender-mixed and older sample.

The study was approved by the local ethics committee (Faculty of Medicine, Ludwig-Maximilians-University, Munich, Germany; registration number: 281-11). All methods were carried out in accordance with the ethical guidelines of the German Psychological Society (DGPs), which are a German adaption of the ethical guidelines (“Ethical Principles of Psychologists and Code of Conduct”) provided by the American Psychological Association (APA) as well as with the Good Clinical Practice (GCP) guidelines. All participants gave their written informed consent for participation in this study. Participants were recruited through student advertisements and flyers. Participants were excluded in case (1) they met the criteria of a psychiatric disorder, measured with the German Version of the Structural Clinical Interview Axis I (SCID-I)^27^ for axis I disorders and the German version of the International Personality Disorder Examination (IPDE)^28^ for axis II disorders or with the German Version of the Structural Clinical Interview Axis II (SCID-II)^27^ for axis II disorders, (2) they had received psychotherapy within the last 10 years, (3) they received psychopharmacological treatment, (4) they had a score > 11 on the Becks Depression Inventory II (BDI-II)^29^, (5) they were pregnant or breast feeding. Additionally, we included only females in experiment 1. These strict inclusion criteria were mainly used as this sample was part of a larger project, which also includes psychiatric samples. A screening of whether participants met the inclusion criteria was conducted via telephone. If the inclusion criteria were met, they were invited for participation in the experiment (see description below). All participants were tested in the laboratory of the Psychiatric hospital of the Ludwig-Maximilians-University. In experiment 1, the experimenter sat in the room next to the testing room in order to take the blood samples. In experiment 2, the experimenter was present during the test situation. After the experiment, participants were thanked, debriefed and received a financial compensation (60 Euros in experiment 1 and 30 Euros in experiment 2). The financial compensation was higher in experiment 1, because the experiment included blood sampling for measuring peripheral levels of oxytocin during the experiment (results will be published elsewhere).

### Measurements

#### A modified Cyberball paradigm

**Figure 1:**
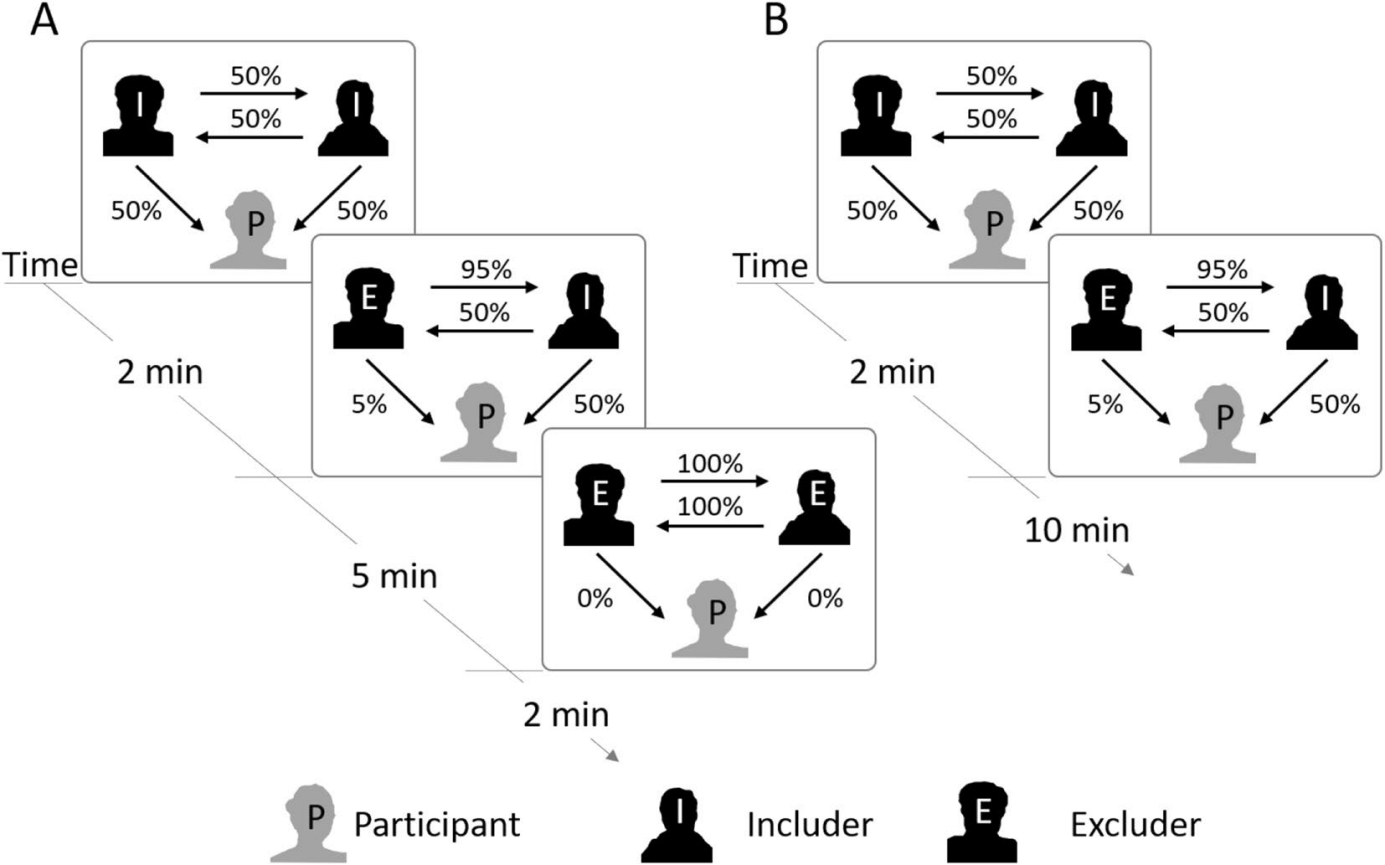
(**A**) Design of the modified Cyberball paradigm in experiment 1. Participants play two min social inclusion (baseline period; 50% ball tosses from both players), followed by five min partial exclusion (5% ball tosses from the excluding player and 50% ball tosses from the including player) and two min total exclusion (no ball tosses from both players). (**B**) Design of the modified Cyberball paradigm in experiment 2. Participants play two min social inclusion (baseline period; 50% ball tosses from both players), followed by 10 min partial exclusion (5% ball tosses from the excluding player and 50% ball tosses from the including player). Participants in the control condition (not presented here) play 12 min social inclusion (50% ball tosses from both players).

We used a modified version of the original Cyberball paradigm, aiming to increase credibility of the experimental setup*.* Participants were either asked to provide a personal photo or a photo was taken prior to the experiment. The photo was uploaded on a computer and later presented during the experiment. Participants always played with co-players of the same gender. The position (right or left) and assignment of photos as including or excluding player were randomized among participants. A cover story claimed that the game would be played with two other co-players on the internet. Before and after playing the Cyberball paradigm, all participants were asked to rate the other two players on computerized visual analogue scales (VAS) with respect to attractiveness, dominance, aggressiveness, sympathy and trustfulness. This allows to control for personal attitudes and to measure the effects of Cyberball on sympathy with the other players. The new Cyberball game of experiment 1 itself consisted of three parts: (1) two min inclusion, during which participants received 50% ball tosses from both players, (2) five min partial exclusion, during which participants received 50% ball tosses from the including and 5% ball tosses from the excluding player, (3) five min total exclusion, during which participants did not receive any ball tosses. The design of the modified paradigm is depicted in Fig. 1A.

To replicate our findings from experiment 1 in a larger, gender-mixed and older sample and to gain further insight into the dynamics of the behavioral response on social exclusion, we conducted a second, enhanced experiment. We adjusted the design of experiment 2 as follows (see Fig. 1B): (1) a control condition was added (i.e., 12 min playing the inclusion condition), and participants played the two conditions in randomized order. (2) Different faces were chosen for both conditions to prevent carry-over effects. (3) We extended the partial exclusion period from five to ten minutes in order to study behavioral changes over a longer period of time to study immediate as well as late effects, and (4) we removed the total exclusion period. This resulted in the following experimental design: (1) experimental condition (partial social exclusion): participants played two min social inclusion (50% ball tosses from both players), followed by ten min partial exclusion (50% ball tosses from including player and 5% ball tosses from excluding player). (2) Control condition (social inclusion): participants played 12 min of social inclusion (50% ball tosses from both players). Conditions were presented in randomized order with a 10 min break between conditions, in which participants were allowed to leave the laboratory.

#### Emotional reactions and ratings of the player

We measured emotional reactions to the exclusion situations by using a modified version of the 14-item emotion scale^30^, which has been adapted for the Cyberball paradigm^31,32^. In this questionnaire participants rate their current emotions (e.g. anger, sadness, happiness) on 7-point Likert Scales before and after the game. According to Jobst et al.^23,24^, emotions were grouped into positive emotions, other-focused negative emotions and self-focused negative emotions. Participants also rated the other players regarding sympathy, attractiveness, trustfulness, dominance and aggressiveness by simply sliding a bar on a VAS displayed on the computer screen, indicating the degree of the rated trait.

### Analyses

#### Behavioral responses

Minute-wise, individual passing preferences *PP(t)* were computed by means of the following formula

1 $$PP(m)={\left(\frac{p2e-p2i}{p2e+p2i}\right)}_{m}$$

with min *m*, ball tosses from participant to excluding player *p2e* or to the including player *p2i*. A positive *PP(m)* value means a passing tendency towards the excluding and a negative value toward the including player. If there is no preference, the value is zero.

Data collected during experiment 1 (7 *PP’s*: 2 no exclusion/5 partial exclusion) were then analyzed using Linear Mixed Model (LMM) analysis. In this analysis, the *PP’s* are considered as nested within subjects. Interindividual differences in baseline playing behavior were considered by including the intercept as a random factor. Model coefficients were computed using restricted maximum likelihood estimation (REML). Parameter estimation used Satterthwaite approximation of degrees of freedom. Pairwise comparisons of minute specific PP’s were carried out using Tukey corrected contrasts. Effect sizes were reported as Cohens *d* for differences in passing behavior, and as Ω^2^^33^ for the amount of variance explained by each factor. Effects were considered to be significant at α = 0.05.

Data collected during experiment 2 (2 × 12 PP’s: 2 no exclusion/10 partial exclusion and 12 inclusion in control task) were used to investigate response dynamics. To this end we used exploratory information from experiment 1 and employed a piece-wise LMM with three subsequent time bins. This allows testing changes in playing behavior over the course of the following subsequent predefined periods: period 1, the *no exclusion phase* starts with the first ball toss and ends by the onset of partial exclusion after 2 min. The end of period 2, the *phase of immediate behavioral response*, was defined by the mean turning point in passing behavior after 2 min of partial exclusion (maximum in the group-mean *PP-*time-series) observed in experiment 1. Period 3, the *phase of late response extinction*, comprised the remaining time. The coding scheme for the specific phases is summarized in Supplementary Table S1. Each period and their interaction with condition were included as fixed factors. Effect sizes for differences between conditions within each period were reported as Cohen’s *d* calculated using the formula

2 $$d=\frac{\beta xTime}{{SD}_{RAW}}$$

as recommended by Feingold^35^.

To test whether between-condition differences in slopes within Period 2 and 3 are detectable with a given sample size (N = 94) and significance level (α = 0.05), we conducted a post hoc power analysis. Assuming identical fixed and random-effect structures as well as identical values for remaining fixed effect estimates, residual variation, and intercept variation as in the original model we computed statistical power over a continuous effect size spectrum in 1000 iterations of Monte-Carlo simulation. Effect sizes for slope differences were defined as Cohen’s d as suggested by Westfall et al.^34^. Results of this power analysis are presented in Supplementary Table S2 and Supplementary Figure S1. To gain more insight into effects rather caused by the set-up of a three players ball game than social exclusion (especially a possible bias in passing behavior), we subtracted no return passes (ball tosses that were forwarded to the next player) from return passes (ball tosses that were returned to the player, who passed the ball) in each minute and re-ran the piecewise LMM of experiment 2 as outlined above. Consequently zero represents the tendency of equal ball tosses to both players, a positive difference represents the tendency to return the ball to the player from whom the participant received the ball, and a negative difference represent the tendency to forward the ball to the next player. For minutes, where participants did not receive a ball from one of the players (in particular the excluder, who passes the ball to the participant merely with ~ 4% chance), the parameter value (which must equal zero, when neither return nor non-returned passes can be tossed) was set to missing.

#### Emotional reactions and ratings of the players

To analyze changes in ratings of emotions and attitudes to players, we used LMM. Restricted maximum likelihood estimation (REML) was applied to compute regression parameters. Fixed effects included time (pre, post) in experiment 1 and time (pre, post), condition (experimental/exclusion versus control/inclusion), and the time × condition interaction in experiment 2. Random effects were addressed identically as in the behavioral analysis.

## Results

### Sample

68 healthy females (mean age 24.76 ± 4.05 years) participated in experiment 1 and 94 healthy participants (mean age 34.50 ± 12.08 years; 48 females) participated in experiment 2 (demographic data for both experiments are presented in Table 1). Both samples were collected independently from each other and there was no overlap in participants.

**Table 1:** Demographic characteristics.

Characteristic	Experiment 1	Experiment 2
*N*	68	94
Females	68	48
Age (means, SD)	24.76 (4.05)	34.5 (12.08)
Years of schooling (means, SD)	17 (2.63)	16.86 (2.81)
Marital status, in relationship (percentage)	40	53

*SD* standard deviation.

### Playing behavior

#### Experiment 1

**Figure 2:**
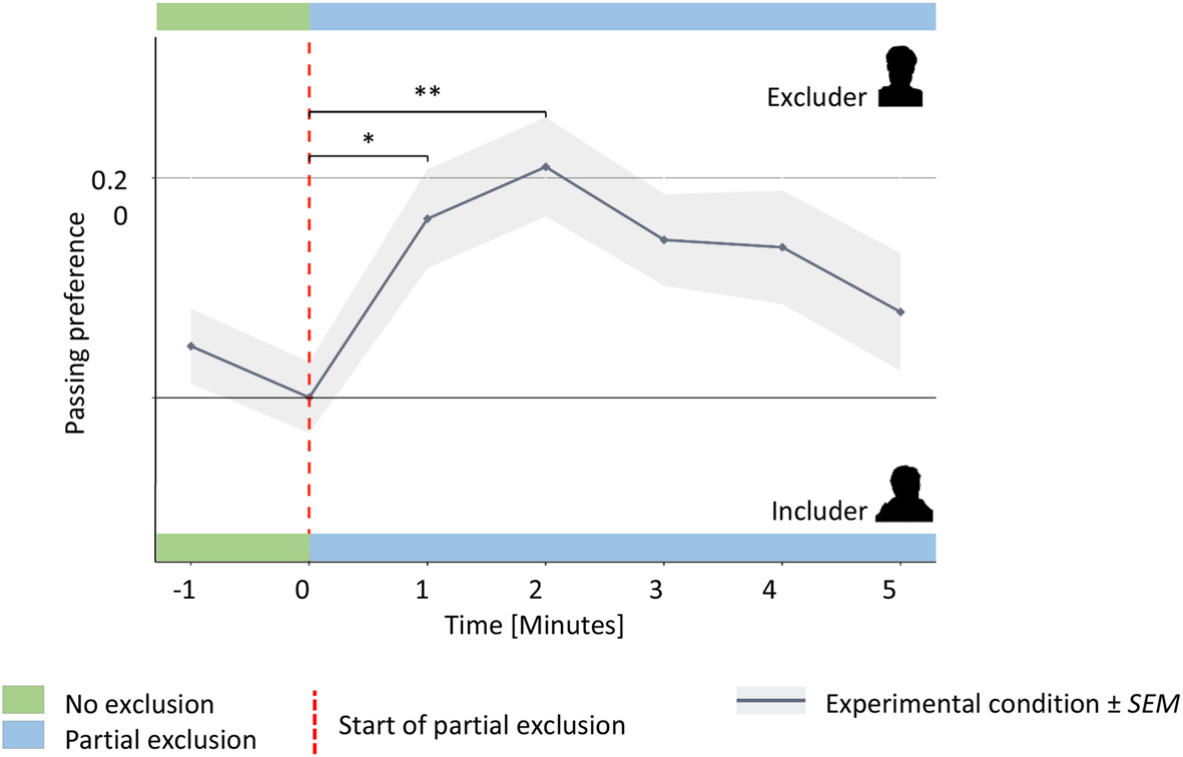
Experiment 1—Trajectory of passing preferences. (**B**) Mean passing trajectories over the course of experiment 1. Passing preferences (PP) were calculated for each minute according to formula [1]. The gray shaded area shows the associated standard errors of means. Dashed red line represents transition from period of no exclusion to partial exclusion period. Asterisks representsss significant Tukey corrected differences in mean passing tendency. *p < 0.05; **p< 0.01; ***p< 0.001.

The minute-wise passing behavior can be found in Supplementary Table S3. We found a significant time effect (F_(6,396.54)_ = 3.59, p = 0.002, Ω^2^ = 0.32) reflecting differences in passing behavior between minutely measurements. Tukey-adjusted pairwise comparisons revealed significant differences in passing behavior between the end of the no exclusion phase and the first (t_(399.44)_ = 3.06, p = 0.038, d = 0.51 95% CI 0.16–0.85) and second min (t_(399.02)_ = 3.85, p = 0.003, d = 0.65 95% CI 0.30–1.0) of the partial exclusion phase. The results show that during the initial inclusion phase the ball was passed equally as often towards the includer and excluder. This behavior changed during the first two min of partial exclusion with more ball tosses directed towards the excluding player (Fig. 2). This behavior disappeared after two min and turned to a similar playing behavior as in the initial inclusion phase. The complete results of the pairwise comparisons between time points are summarized in Supplementary Table S4.

#### Experiment 2

**Figure 3:**
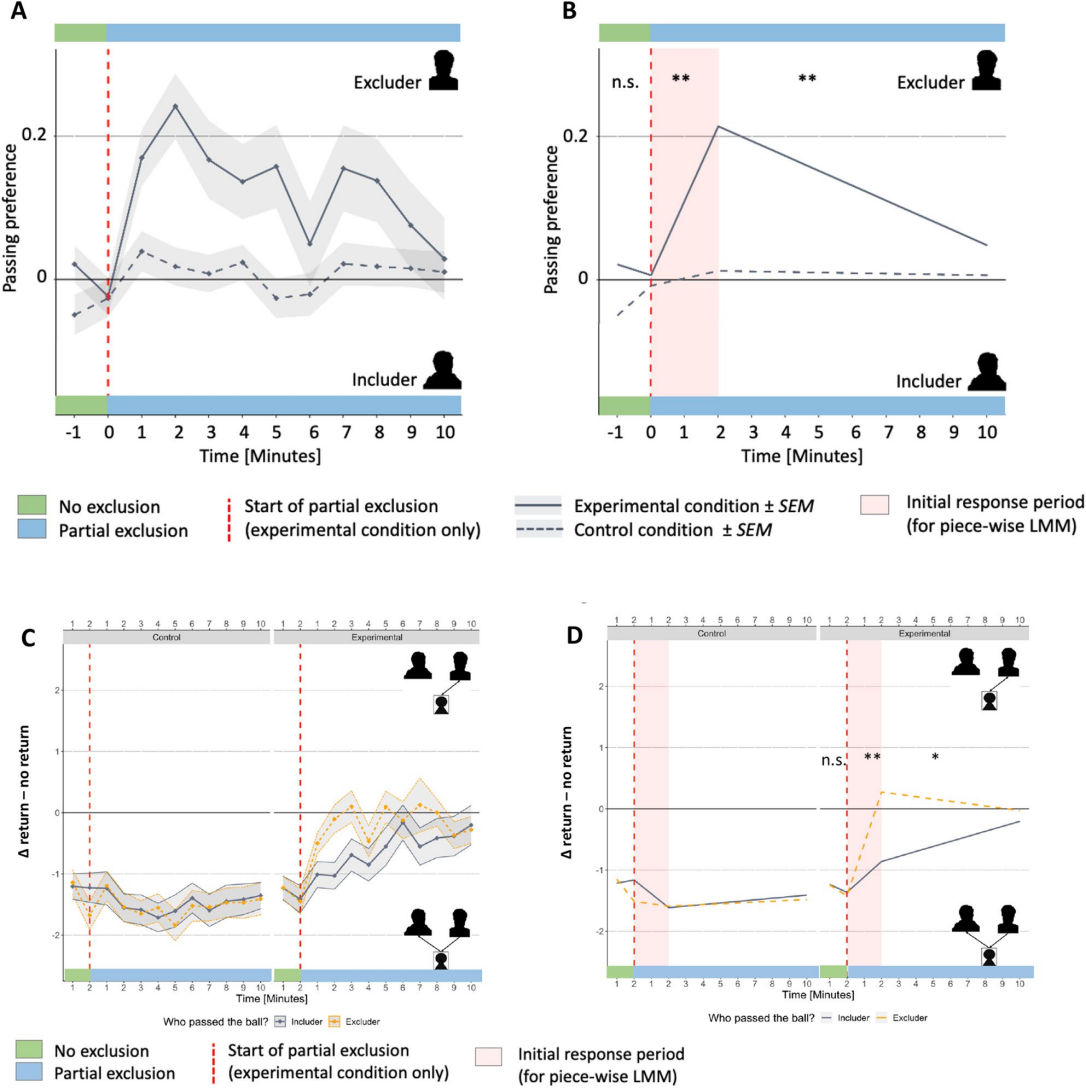
Experiment 2. (**A**) Mean passing trajectories over the course of experiment 2. Passing preferences (PP) were calculated for each minute according to formula [1]. The gray shaded area shows the associated standard errors of means. Dashed red line represents transition from period of no exclusion to partial exclusion period. (**B**) Predicted values of piecewise (Linear Mixed Model) LMM. (**C**) Δ return passes—no return passes were calculated for each minute. (**D**) Predicted values of piecewise LMM. 0 represents a balanced return versus no return behavior, values < 0 represent more return playing, values > 0 represent forwarding playing. Asterisks represent significant period × condition interactions. *p< 0.05; **p < 0.01; ***p < 0.001.

The piecewise LMM showed significant differences between experimental and control condition in passing behavior during the second, immediate response period (*F*_(1,2143.3)_ = 9.40, *p* = 0.002, *d* = 0.75, 95% CI 0.27–1.22) and the third, late extinction period (*F*_(1,2143.3)_ = 8.85, *p* = 0.003, *d* = − 0.61, 95% CI − 1.01 to − 0.21, but not for the first, no exclusion period (*F*_(1,2143.3)_ = 0.62, *p* = 0.433, *d* = − 0.21, 95% CI − 0.75 to 0.32). This dynamic and transient change in playing behavior, which has also been observed in experiment 1, appears to be an elementary response to social exclusion, and which occurred only in the experimental condition but not in the control condition. Participants reacted with an initial, about 2 min lasting tendency to pass the ball towards the excluder rather than the includer, followed by a slow progression towards a balanced playing behavior (equal number of tosses towards both players). Table 2 and Fig. 3A,B present the results of experiment 2.

**Table 2:** Results of piecewise Linear Mixed Models (LMM; experiment 2).

Period	Time interval (minutes)	Slope		Period × Condition		
		Control	Experimental	F value	p value	Effect size (d)
1: no exclusion	[1,2]	0.041	− 0.015	0.62	0.433	− 0.21 (− 0.75 to 0.32)
2: immediate	[3,4]	0.010	0.104	9.40	0.002**	0.75 (0.27 to 1.22)
3: extinction	[5,10]	− 0.001	− 0.021	8.85	0.003**	− 0.61 (− 1.01 to − 0.21)

Passing preferences were calculated for each minute according to formula [1]; slope represents group-specific change in passing tendency within respective period; significant interaction effect represents difference in group-specific slopes within respective period; effect size calculated as Cohen’s d according to formula [2]. *p < 0.05 **p< 0.01; ***p< 0.001.

In the experimental condition, we found a significant period × passer interaction for the second, immediate response period (F _(1,1425)_ = 8.23, *p* = 0.004; *d* = 0.55, 95% CI 0.17–0.93) and the third, late extinction period (F _(1,1430)_ = 4.05, *p* = 0.04; *d* = 0.46, 95% CI − 0.91 to − 0.01, indicating that participants returned the ball more often after having received it from the excluding player. This period × passer interaction was neither significant in the first, no exclusion phase, nor in the control condition (all *p* > 0.05). Table 3 and Fig. 3C,D show the results of this additional analysis.

**Table 3:** Piecewise regression results for return vs. no return passing.

Phase	Slope		Period × Passer		Effect size (d)
	Includer	Excluder	F value	p value	
**Experimental**					
1: no exclusion	− 0.130	− 0.181	0.02	0.898	− 0.03 (− 0.46 to 0.4)
2: immediate	0.252	0.848	8.23	0.004**	0.55 (0.17 to 0.93)
3: extinction	0.082	− 0.038	4.05	0.044**	− 0.46 (-0.91 to -0.01)
**Control**					
1: no exclusion	0.050	− 0.363	1.31	0.253	− 0.21 (− 0.57 to 0.15)
2: immediate	− 0.225	− 0.040	1.46	0.226	0.17 (− 0.11 to 0.45)
3: extinction	0.026	0.014	0.12	0.725	− 0.04 (− 0.28 to 0.19)

To test specific effects of sociodemographic variables, we added three-way interaction effects for each period with condition as well as age and gender. While the two-way interaction between condition and period 2 and 3, respectively remained significant (gender included: condition × period 2: F _(1,2136)_ = 9.53, *p* = 0.002; condition × period 3: F _(1,2136)_ = 8.95, *p* = 0.003; age included: condition × period 2: F _(1,2136)_ = 9.40, *p* = 0.002; condition × period 3: F _(1,2136)_ = 8.88, *p* = 0.003), we did not find significant three-way interactions for age in period 2 (F _(1,2136)_ = 0.02, *p* = 0.90) and 3 (F _(1,2136)_ = 1.89, *p* = 0.17) and for gender in period 2 (F _(1,2136)_ = 1.19, *p* = 0.28) and 3 (F _(1,2136)_ = 0.39, *p* = 0.53).

### Emotional reactions and ratings of other players

In experiment 1, we found a significant increase in other-focused negative emotions (*t*_(67)_ = 5.55, *p* < 0.001), self-focused negative emotions (*t*_(65)_ = 2.02, *p* = 0.047) and a significant decrease in positive emotions (*t*_(66)_ = − 4.9, *p* < 0.001). In experiment 2, emotional changes in the experimental condition were comparable to the results of experiment 1, however, other-focused negative emotions also increased in the control condition (*t*_(278)_ = 3.94, *p* = 0.004). The interaction showed that changes in the experimental group differed from the control condition for self-focused negative emotions (*F*_(1,278)_ = 4.21, *p* = 0.041) and other-focused negative emotions (*F*_(1,278)_ = 15.54, *p* < 0.001), but not for positive emotions (*F*_(1,278)_ = 2.90, *p* = 0.090).

**Figure 4:**
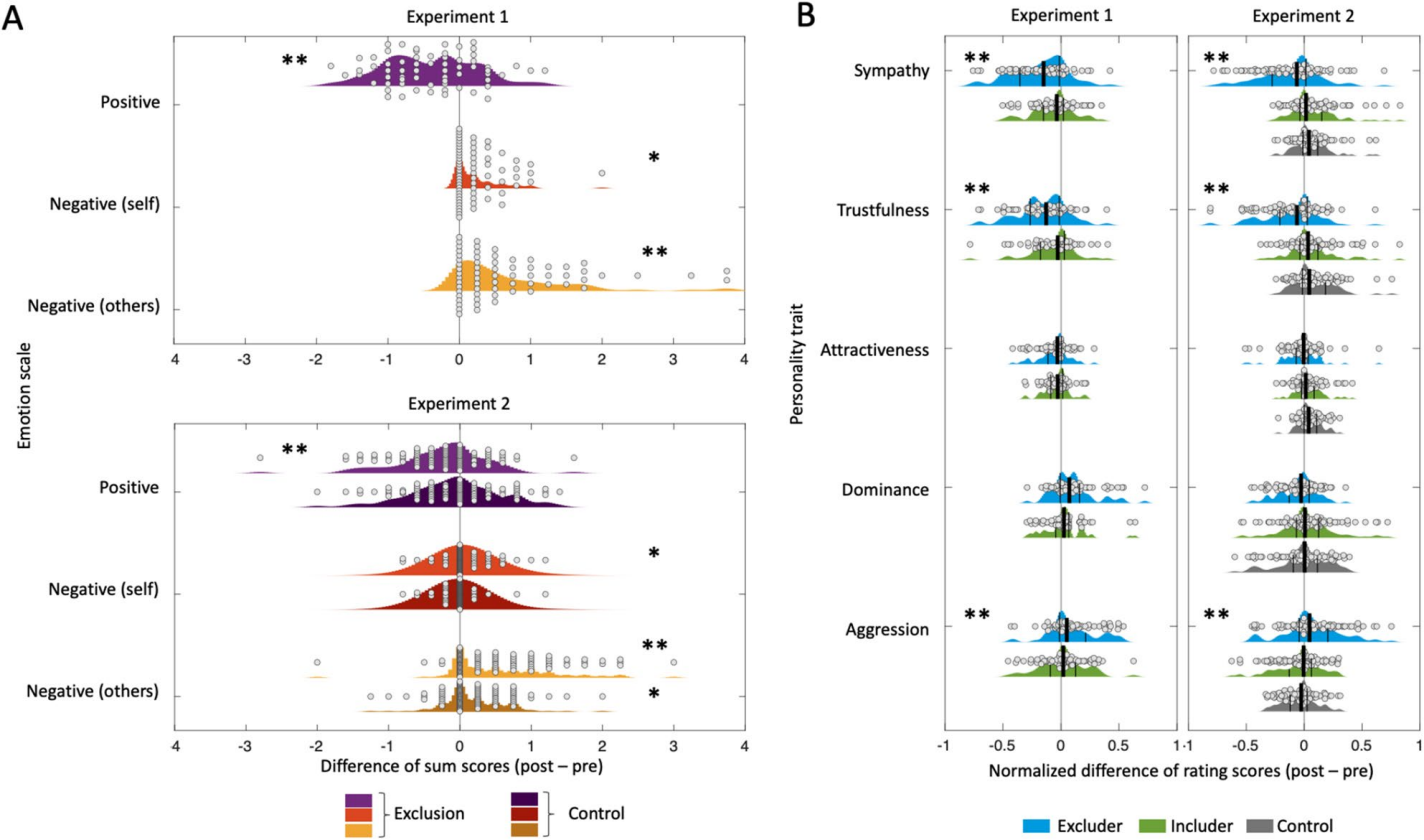
Emotional reactions and ratings of other players. Displayed are individual values (circles) and associated probability densities for (**A**) Emotions threat through social exclusion: post–pre gaming differences in emotional sub scale sum scores for experiment 1 (upper three rows) and experiment 2 (lower six rows). Results from control condition for experiment 2 are displayed in darkened colors. (**B**) The effect of social exclusion on personality trait ratings. Results for control condition in experiment 2 are displayed in gray. Additionally, the median (thick black line) as well as the 25 and 75% percentiles (thin black lines) are shown. Asterisks represent significant differences between pre and post ratings/sum scores. *p < 0.05; **p < 0.01.

In experiment 1, participants rated the excluding player as more dominant (*t*_(65)_ = 4.34, *p* < 0.001), more aggressive (*t*_(65)_ = 3.93, *p* < 0.001), less sympathetic (*t*_(66)_ = − 6.54, *p* < 0.001) and less trustful (*t*_(66)_ =  − 6.12, *p* < 0.001) after the game when compared to their initial pre-ratings. Interestingly, similar results were found for the ratings of the including player concerning sympathy (*t*_(66)_ =  − 3.25, *p* = 0.002), trust (*t*_(66)_ =  − 3.00, *p* = 0.004) and attractiveness (*t*_(64)_ =  − 3.02, *p* = 0.004), whereas no changes occurred concerning dominance (*t*_(65)_ = 1.08, *p* = 0.283) and aggressiveness (*t*_(66)_ = 0.85, *p* = 0.397). In experiment 2, our results show a significant difference in changes of ratings of the excluder between the experimental and the control group (all *p* < 0.05). Means, standard deviations and test statistics are shown in Table 4 and results are presented in Fig. 4.

**Table 4:** Emotional reactions and ratings of other players.

	Experiment 1				Experiment 2									
					Control				Experimental				Control vs. experimental	
	Pre	Post	Slope	p	Pre	Post	Slope	p	Pre	Post	Slope	p	F	p
**Sympathy (E)**	0.66 (0.16)	0.47 (0.21)	− 0.19	< 0.001***	0.61 (0.23)	0.67 (0.18)	0.06	0.012*	0.58 (0.23)	0.47 (0.26)	− 0.11	< 0.001***	25.34	< 0.001***
**Trustfulness (E)**	0.66 (0.19)	0.5 (0.18)	− 0.16	< 0.001***	0.58 (0.22)	0.65 (0.17)	0.08	< 0.001***	0.58 (0.21)	0.47 (0.24)	− 0.11	< 0.001***	38.81	< 0.001***
Attractiveness (E)	0.54 (0.17)	0.48 (0.18)	− 0.06	< 0.001***	0.47 (0.24)	0.51 (0.22)	0.04	0.024*	0.47 (0.24)	0.45 (0.24)	− 0.02	0.295	5.53	0.019*
Dominance (E)	0.38 (0.21)	0.47 (0.24)	0.10	< 0.001***	0.39 (0.22)	0.33 (0.2)	− 0.06	0.013*	0.4 (0.23)	0.43 (0.25)	0.03	0.215	6.97	0.009**
**Aggression (E)**	0.25 (0.21)	0.35 (0.25)	0.10	< 0.001***	0.28 (0.22)	0.23 (0.18)	− 0.05	0.033*	0.32 (0.22)	0.38 (0.24)	0.07	0.008**	11.68	0.001***
*Sympathy (I)*	*0.69 (0.14)*	*0.62 (0.17)*	*− 0.07*	0.002**	*0.62 (0.23)*	*0.67 (0.19)*	*0.06*	0.011*	*0.61 (0.22)*	*0.68 (0.19)*	*0.06*	0.003**	*0.07*	*0.787*
*Trustfulness (I)*	*0.67 (0.15)*	*0.6 (0.17)*	*− 0.07*	0.004**	*0.57 (0.23)*	*0.63 (0.2)*	*0.06*	0.009**	*0.58 (0.21)*	*0.64 (0.2)*	*0.06*	0.011*	*0.00*	*0.950*
*Attractiveness (I)*	*0.56 (0.16)*	*0.52 (0.17)*	*− 0.04*	0.004**	*0.45 (0.23)*	*0.5 (0.21)*	*0.05*	0.003**	*0.48 (0.22)*	*0.52 (0.23)*	*0.04*	0.044*	*0.50*	*0.481*
*Dominance (I)*	*0.36 (0.19)*	*0.38 (0.22)*	*0.02*	*0.283*	*0.37 (0.21)*	*0.33 (0.21)*	*− 0.04*	*0.116*	*0.38 (0.23)*	*0.37 (0.19)*	*− 0.01*	*0.758*	*0.81*	*0.369*
*Aggression (I)*	*0.24 (0.19)*	*0.26 (0.2)*	*0.02*	*0.397*	*0.26 (0.21)*	*0.24 (0.2)*	*− 0.03*	*0.269*	*0.29 (0.22)*	*0.25 (0.19)*	*− 0.04*	*0.063*	*0.28*	*0.596*
**Self-focused negative emotions**	1.21 (0.33)	1.31 (0.48)	0.11	0.047*	1.15 (0.35)	1.13 (0.33)	− 0.02	0.417	1.16 (0.37)	1.22 (0.42)	0.06	0.038*	4.21	0.041*
Positive emotions	3.74 (0.94)	3.37 (1.04)	− 0.39	< 0.001***	3.67 (1.08)	3.6 (1.2)	− 0.07	0.286	3.64 (1.09)	3.41 (1.1)	− 0.24	0.001***	2.90	0.090
**Other-focused negative emotions**	1.21 (0.33)	1.83 (0.93)	0.63	< 0.001***	1.31 (0.52)	1.5 (0.56)	0.19	0.004**	1.34 (0.48)	1.88 (0.9)	0.55	< 0.001***	15.54	< 0.001***

Descriptive statistics and results of Linear Mixed Models (LMM) analyses.

*E* excluder, *I* includer; pre and post values reported as mean (SD); significance of pre-post LMM slope coefficients computed using Wald-Tests; significance of LMM factor interaction computed using Type III analysis of variance with Satterthwaite's method, personal characteristics rating for includer are highlighted in italics. Exclusion induced changes in the ratings of personal characteristics and emotions that show similar effects in both experiments and can thus be considered as replicated, are highlighted in bold.

*p < 0.05; **p < 0.01; ***p < 0.001.

## Discussion

In this study, we used a modified version of the Cyberball game with a partial exclusion condition, which allows to measure immediate behavioral responses to social exclusion and model these responses over time. Although partial exclusion has been studied before^36^, to the best of our knowledge, this study is the first attempt at modeling people’s dynamic behavioral reactions to social exclusion, and to gain deeper insight into phase-specific responses, according to behavioral changes over time. Additionally, we assessed emotional changes and changes in ratings of the other players.

We observed an immediate behavioral response within the first two minutes of partial exclusion, i.e., the participants played more often to the excluder rather than to the includer. In contrast, baseline inclusion phases in both experiments as well as the control condition (inclusion only) in experiment 2 showed a preferred tendency to pass the ball in a “no return” manner (i.e. not returning the ball to the player from whom the ball was received). This initial behavior disappeared during partial exclusion, and the amount of return passes to the excluder increased. This phase was followed by a late period, characterized by an extinction of the immediate response after partial exclusion, where playing behavior qualitatively returned to baseline (i.e., no preference towards one of the players). Interestingly, within the exclusion period, also the number of return passes to the includer increased linearly, until at the end of this period neither a “return” nor a “no return” behavior was observable any more.

To the best of our knowledge, this dynamic response, which was first observed in experiment 1 and replicated in experiment 2, has not been reported before in social exclusion studies. Particularly, the immediate response may represent a characteristic initial reaction to social exclusion. During partial exclusion, participants never preferred playing to the including player at any time point. This was observed already in experiment 1 (5 min partial exclusion), but also found in experiment 2 where the partial exclusion period was extended to 10 min. Consequently, we did not find a “behavioral cross-over- point”, meaning that participants returned to their baseline behavior after several minutes but did not start to toss more balls towards the including player. In other words, we did not find a behavioral change that would indicate that participants start to exclude the excluding player and solely connect with the including player. Furthermore, distinct differences in behavioral and emotional responses between experimental and control conditions of experiment 2 support the robustness of the effect and underlines the idea that this effect seems to be a specific reaction to social exclusion, probably aiming to regulate emotions and emotional well-being as well as to strive for re-inclusion.

Our analysis of return vs no return passes show that the behavioral responses to social exclusion cannot solely be attributed to the fact, that participants receive more balls from the including player and may tend to simply forward the ball to the next player. We therefore argue that the reported effects represent a behavioral response resulting from the experience of social exclusion. However, different interpretations of this finding can be discussed: first, the observed immediate behavioral response (i.e., playing to the excluder) refers to the idea that participants try to reconnect with the excluding individual^7^ This would indicate prosocial tendencies towards repair of broken cooperation. Such an interpretation is supported by theories like the social reconnection hypothesis^7^ and the SBT^9^, by which social connection is less energy consuming than open conflicts and participants may strive for group cohesiveness. Alternatively, the immediate response may represent an attempt to intensify the interpersonal contact with the excluder, by making him aware of uncooperative (excluding) behavior in order to reestablish cooperation. Another concept that may underlie the behavioral response on an individual basis, is the idea of forgiveness. This would mean, that the excluded participant aims to signal the excluding player that he “forgives” him and consequently aims to repair the broken relationship. Because the immediate response does not lead to behavioral changes on the excluder’s site, this behavior weakens and eventually disappears completely (behavioral extinction), possibly indicating the attempt to find a behavioral balance that is as least distressing as possible. Alternatively, participants may show the reported behavior in order to maintain social norms (e.g., equality norm) within a group. Our observation of an unbiased passing behavior at the end of the exclusion period (regarding the absolute number of passes as well as the return and no return passes), is in line with this interpretation.

As the effects do not differ between the two samples and three-way interactions did not reach significance whereas the significance of the two-way interactions did not change, we assume that differences in age and gender had no influence on the intensity or temporal characteristics of the behavioral response points towards a robust and reliable behavioral effect. This result supports the findings of a meta-analysis of 120 Cyberball studies, which also did not find moderation effects for age and gender^19^. According to the multi-motive model by Smart Richman and Leary^2^, our results point towards the idea that in the beginning participants expected the exclusion to be of high costs, expected relational repair and/or attributed a high value to the relationship. As the behavior changed over time, it could be that also motives changed during the exclusion process. Adding changes over time (e.g., as proposed by Williams^3^) to the model, would enable to picture the complexity of the process during social exclusion.

Although we could replicate the general behavioral pattern observed in experiment 1, the results do not indicate that all participants showed the same behavior. This is in line with the theoretical model proposed by Smart Richman and Leary^2^ and is very likely a result of different motives that are rooted in different biographical experiences and the associated (probably learned) social habits. However, it remains unclear, which individual factors (e.g., attachment style, rejection sensitivity) may influence or even cause a certain behavioral pattern.

Striking evidence exist concerning the effects of social exclusion on emotions. While Blackhart et al.^14^ concluded in their review that the effects of social exclusion eliminate positive emotions but do not necessarily increase negative emotions, other studies show effects on both positive and negative emotions^19,20^. Our results support the latter as they show a reliable increase in negative emotions and reduction of positive emotions, following social exclusion. The results of experiment 2 confirm the hypothesis, that experimental partial social exclusion induces an increase in negative emotions.

Beside the replication of the immediate behavioral response, we found a reliable decrease in sympathy and trustfulness ratings as well as an increase in aggressiveness ratings of the excluding player. The effects remained even if they were compared to the control condition in experiment 2. Moreover, the decrease in excluders sympathy und trustfulness are in line with recently published findings from a comparable Cyberball study by Roayaee et al.^37^. Interestingly, the increase in sympathy, trustfulness and attractivity rating for the includer observed in experiment 1, did not survive this control for probably unspecific task effects. Considering the fact, that our findings regarding a decrease of positive emotions in experiment 1 could not be confirmed in the second experiment as the interaction effect for positive emotions was not significant, it is likely, that behavioral as well as emotional responses to social exclusion evoke a strong negative bias in the attitudes towards self and others.

However, as current emotional reactions were assessed retrospectively as we measured them before and after—but not during—the experiment and participants were not asked about their emotions during the game, they should not be interpreted as an indication of the reflexive stage, because they may be influenced by the factor time, which underlies retrospective processes (e.g., cognitive bias). Coping strategies (e.g. cognitive restructuring, emotion regulation strategies) may therefore play a central role concerning the effects of social exclusion.

### Limitations

The following limitations should be mentioned: (1) most participants were students, representing a specific subgroup of the general population. However, even though the sample in experiment 2 was more diverse, results from experiment 1 could be replicated. (2) As the duration of the paradigm was 12 min in experiment 2, it could be speculated that participants may have felt bored and that this may have influenced their behavior and emotional ratings. Variations of experiment duration should therefore be tested in future studies. (3) Emotions were measured before and after the game but no measure was included that assesses emotional reactions directly during the social exclusion situation, e.g. with high frequency experience sampling. (4) The tailing period of total exclusion in experiment 1, may have influenced the ratings for the mainly including player. Therefore, the between-experiment comparability of our findings in this respect is very limited. (5) Although we were able to replicate the main findings from experiment 1 in experiment 2, the experimental setup differed between the two studies (e.g., blood samples were taken in experiment 2), which may have influenced the results.

### Future research

The paradigm proposed here already includes a dynamic model of two phases in the behavioral reaction to partial social exclusion. However, we regard this hypothetical model as preliminary, and future studies should (1) replicate our findings in larger samples, (2) test its test–retest reliability, (3) further optimize the model in mathematical terms, and (4) investigate its neurobiological underpinnings. Replication studies should systematically vary exclusion parameters, e.g., length and extent of partial exclusion (i.e., here the percentage of ball tosses from both players). (5) Based on the assumption that the need of belonging is especially important for relationships with meaningful people and to a lesser extend with unknown individuals^6^, future studies may also investigate whether behavioral responses depend on the existing interpersonal relationship between participants and co-players (e.g., strangers as co-players versus people with close interpersonal relationships: in-group/out-group members). Finally, although we have argued against the idea that playing behavior during partial exclusion represents an automatic behavioral reaction, namely passing the ball to the next player, rather than a specific effect of social exclusion, we highly recommend to conduct further experiments varying the number of players in order to disentangle overlapping and interacting principles of collaboration.

## Supplementary Information

Supplementary Information.

## Data Availability

The datasets generated during and/or analysed during the current study are available from the corresponding author on reasonable request.
