# Supplementary material for: Dynamics of the immediate behavioral response to partial social exclusion
Source: Sci Rep. 2021 Jan 20;11:1853. doi: 10.1038/s41598-020-80039-0 (PMC7817687; doi:10.1038/s41598-020-80039-0)
Supplement: Supplementary file 1 — Supplementary Information. [file 41598_2020_80039_MOESM1_ESM.docx]

**Title:** Dynamics of the immediate behavioral response to partial social exclusion

Dewald-Kaufmann, J.F.*,°, ^1,2^, Wüstenberg, T.*^3,4^, Barton, B.B.^1^, Goerigk, S.^1,2,5^, Reinhard, M.A.^1^, Musil, R.^1^, Werle, J. ^1^, Falkai, P. ^1,^ Jobst, A.*^1^, Padberg, F.*^1^

***** Authors contributed equally

° Corresponding author (e-mail: julia.dewald_kaufmann@med.uni-muenchen.de)

^1^Department of Psychiatry and Psychotherapy, University Hospital, Ludwig-Maximilians-University Munich, Germany

^2^Hochschule Fresenius, University of Applied Sciences, Munich, Germany

^3^Department of Psychiatry and Psychotherapy, Charité Campus Mitte, Charité – Universitätsmedizin Berlin, Germany

^4^ Department of Clinical Psychology and Psychotherapy, Ruprecht-Karls-University Heidelberg, Heidelberg, Germany

^5^Department of Psychological Methodology and Assessment, Ludwig-Maximilians-University Munich, Germany

**Supplementary Table 1:** Coding scheme of Linear Mixed Models (LMM) time bins in experiment 2.

| **Measurement** | **Period 1** | **Period 2** | **Period 3** |
| --- | --- | --- | --- |
| NEx [Min: 1] | 0 | 0 | 0 |
| NEx [Min: 2] | 1 | 0 | 0 |
| PEx [Min: 1] | 1 | 1 | 0 |
| PEx [Min: 2] | 1 | 2 | 0 |
| PEx [Min: 3] | 1 | 2 | 1 |
| PEx [Min: 4] | 1 | 2 | 2 |
| PEx [Min: 5] | 1 | 2 | 3 |
| PEx [Min: 6] | 1 | 2 | 4 |
| PEx [Min: 7] | 1 | 2 | 5 |
| PEx [Min: 8] | 1 | 2 | 6 |
| PEx [Min: 9] | 1 | 2 | 7 |
| PEx [Min: 10] | 1 | 2 | 8 |

Note: NEx no exclusion; PEx partial exclusion; Min minute

**Supplementary Table 2:** Power analysis (experiment 2)

| **Effect size** | **Power Period 2 [%]** | **Power Period 3 [%]** |
| --- | --- | --- |
| 0.00 | 5.7 | 4.1 |
| 0.05 | 5.8 | 4.7 |
| 0.10 | 7.6 | 6.2 |
| 0.15 | 10.4 | 9.4 |
| 0.20 | 14.6 | 15.0 |
| 0.25 | 20.7 | 20.4 |
| 0.30 | 26.7 | 27.9 |
| 0.35 | 33.1 | 36.5 |
| 0.40 | 40.6 | 45.7 |
| 0.45 | 47.4 | 55.7 |
| 0.50 | 56.1 | 66.6 |
| 0.55 | 64.1 | 74.3 |
| 0.60 | 71.1 | 80.7 |
| 0.65 | 76.9 | 86.8 |
| 0.70 | 82.5 | 91.4 |
| 0.75 | 87.7 | 94.9 |
| 0.80 | 90.1 | 96.9 |
| 0.85 | 93.5 | 98.0 |
| 0.90 | 95.7 | 99.0 |
| 0.95 | 97.0 | 99.5 |
| 1.00 | 98.4 | 99.7 |

Note: Power computed for period x condition interaction assuming N = 94 and significance level of α= 0.05. Remaining fixed effect estimates, residual variation and intercept variation were held constant as statistical power was estimated for a continuous effect size spectrum in 1000 iterations of Monte-Carlo simulation. Effect sizes for slope differences were defined as Cohen’s d as suggested by Westfall and colleagues (2018).

**Supplementary Table 3:** Means and standard deviation (SD) of passing preferences (PPs) and return versus no return playing

|  |  | Mean (SD) | | | | | | | |
| --- | --- | --- | --- | --- | --- | --- | --- | --- | --- |
|  |  | **Experiment 1** | | | **Experiment 2** | | | | |
|  |  | **Excluder vs. Includer** | | | **Excluder vs. Includer** | | **Return vs. No Return** | | |
|  |  |  |  |  | **Control** | **Experimental** | **Control** | **Experimental** | |
| **Condition** | **Minute** |  |  |  |  |  |  | **Excluder** | **Includer** |
| No Exclusion | 1 | -0.05 (0.28) | | | -0.01 (0.26) | -0.01 (0.26) | -1.17 (1.98) | -1.23 (1.78) | -1.23 (1.84) |
|  | 2 | 0.00 (0.27) | | | -0.03 (0.25) | -0.02 (0.25) | -1.45 (2.26) | -1.45 (2) | -1.41 (2.15) |
| Partial Exclusion | 1 | 0.16 (0.37) | | | 0.04 (0.27) | 0.17 (0.38) | -1.22 (2.45) | -0.5 (1.03) | -1.01 (2.06) |
|  | 2 | 0.21 (0.37) | | | 0.02 (0.26) | 0.24 (0.44) | -1.55 (2.25) | -0.11 (0.99) | -1.03 (2.11) |
|  | 3 | 0.14 (0.34) | | | 0.01 (0.25) | 0.17 (0.53) | -1.62 (2.2) | 0.1 (1.17) | -0.69 (2.54) |
|  | 4 | 0.14 (0.43) | | | 0.02 (0.24) | 0.14 (0.51) | -1.63 (2.24) | -0.47 (0.99) | -0.85 (2.58) |
|  | 5 | 0.08 (0.44) | | | -0.03 (0.27) | 0.16 (0.56) | -1.72 (2.5) | 0.1 (1.18) | -0.55 (3) |
|  | 6 |  |  |  | -0.02 (0.29) | 0.05 (0.57) | -1.46 (2.4) | -0.12 (1.2) | -0.16 (2.77) |
|  | 7 |  |  |  | 0.02 (0.29) | 0.15 (0.58) | -1.57 (2.38) | 0.12 (1.25) | -0.55 (2.95) |
|  | 8 |  |  |  | 0.02 (0.27) | 0.14 (0.56) | -1.46 (2.44) | 0.00 (1.14) | -0.41 (3.09) |
|  | 9 |  |  |  | 0.02 (0.25) | 0.08 (0.58) | -1.44 (2.44) | -0.37 (0.96) | -0.38 (3.13) |
|  | 10 |  |  |  | 0.01 (0.28) | 0.03 (0.56) | -1.38 (2.3) | -0.28 (1.1) | -0.2 (3.12) |

Note: PP Passing preferences, calculated for each minute according to formula [1]; A positive *PP(m)* value means a passing tendency towards the excluding and a negative value toward the including player. If there is no preference, the value is zero; Return passes reflect the tendency to return the ball to the player who passed the ball, no return passes reflect the tendency to forward the ball to the next player; SD standard deviation; Numbers rounded to two decimal places

**Supplementary Table 4:** Pairwise comparisons of mean passing tendency in each minute (experiment 1).

| **Contrast** | **Difference (CI95%)** | **Standard Error** | **Degrees of Freedom** | **T-Value** | **P-Value** | **Effect Size (CI95%)** |
| --- | --- | --- | --- | --- | --- | --- |
| NEx [Min 1] - NEx [Min 2] | 0.05 (-0.11 to 0.21) | 0.05 | 399.78 | 0.90 | 0.973 | 0.17 (-0.17 to 0.52) |
| NEx [Min 1] - PEx [Min 1] | -0.12 (-0.28 to 0.05) | 0.06 | 399.36 | -2.14 | 0.329 | -0.36 (-0.7 to -0.01) |
| NEx [Min 1] - PEx [Min 2] | -0.16 (-0.32 to 0) | 0.05 | 399.78 | -2.92 | 0.056 | -0.5 (-0.85 to -0.15) |
| NEx [Min 1] - PEx [Min 3] | -0.09 (-0.26 to 0.07) | 0.05 | 399.78 | -1.71 | 0.607 | -0.31 (-0.65 to 0.03) |
| NEx [Min 1] - PEx [Min 4] | -0.09 (-0.25 to 0.08) | 0.05 | 399.78 | -1.59 | 0.688 | -0.25 (-0.59 to 0.09) |
| NEx [Min 1] - PEx [Min 5] | -0.03 (-0.19 to 0.13) | 0.05 | 399.78 | -0.52 | 0.999 | -0.08 (-0.43 to 0.26) |
| NEx [Min 2] - PEx [Min 1] | -0.17 (-0.33 to -0.01) | 0.05 | 399.44 | -3.06 | **0.038*** | -0.51 (-0.85 to -0.16) |
| NEx [Min 2] - PEx [Min 2] | -0.21 (-0.37 to -0.05) | 0.05 | 399.02 | -3.85 | **0.003**** | -0.65 (-1 to -0.3) |
| NEx [Min 2] - PEx [Min 3] | -0.14 (-0.3 to 0.02) | 0.05 | 399.02 | -2.63 | 0.119 | -0.47 (-0.81 to -0.12) |
| NEx [Min 2] - PEx [Min 4] | -0.14 (-0.3 to 0.02) | 0.05 | 399.02 | -2.51 | 0.159 | -0.39 (-0.73 to -0.04) |
| NEx [Min 2] - PEx [Min 5] | -0.08 (-0.24 to 0.08) | 0.05 | 399.02 | -1.43 | 0.787 | -0.21 (-0.55 to 0.13) |
| PEx [Min 1] - PEx [Min 2] | -0.04 (-0.2 to 0.12) | 0.05 | 399.44 | -0.78 | 0.987 | -0.13 (-0.47 to 0.21) |
| PEx [Min 1] - PEx [Min 3] | 0.02 (-0.14 to 0.19) | 0.05 | 399.44 | 0.44 | 0.999 | 0.05 (-0.29 to 0.39) |
| PEx [Min 1] - PEx [Min 4] | 0.03 (-0.13 to 0.19) | 0.05 | 399.44 | 0.56 | 0.998 | 0.07 (-0.28 to 0.41) |
| PEx [Min 1] - PEx [Min 5] | 0.09 (-0.07 to 0.25) | 0.05 | 399.44 | 1.64 | 0.658 | 0.21 (-0.13 to 0.55) |
| PEx [Min 2] - PEx [Min 3] | 0.07 (-0.1 to 0.23) | 0.05 | 399.02 | 1.22 | 0.886 | 0.19 (-0.15 to 0.53) |
| PEx [Min 2] - PEx [Min 4] | 0.07 (-0.09 to 0.23) | 0.05 | 399.02 | 1.34 | 0.832 | 0.18 (-0.16 to 0.52) |
| PEx [Min 2] - PEx [Min 5] | 0.13 (-0.03 to 0.29) | 0.05 | 399.02 | 2.42 | 0.191 | 0.32 (-0.02 to 0.67) |
| PEx [Min 3] - PEx [Min 4] | 0.01 (-0.15 to 0.17) | 0.05 | 399.02 | 0.12 | 1.000 | 0.02 (-0.32 to 0.36) |
| PEx [Min 3] - PEx [Min 5] | 0.07 (-0.1 to 0.23) | 0.05 | 399.02 | 1.20 | 0.892 | 0.17 (-0.17 to 0.51) |
| PEx [Min 4] - PEx [Min 5] | 0.06 (-0.1 to 0.22) | 0.05 | 399.02 | 1.08 | 0.933 | 0.14 (-0.2 to 0.48) |

Note: NEx no exclusion; PEx partial exclusion; Min minute; Passing tendency was calculated for each minute as mean throws to includer minus mean throws to excluder. Positive values represent tendency to pass to excluder, negative values represent tendency to pass to includer; P-Values adjusted for multiple comparisons using Tukey method; Effect size calculated as Cohens d; Numbers rounded to two respectively three decimal places; (*) < 0.05 (**) <0.01 (***) <0.001

**Supplementary Figure 1: Power analysis (experiment 2)**


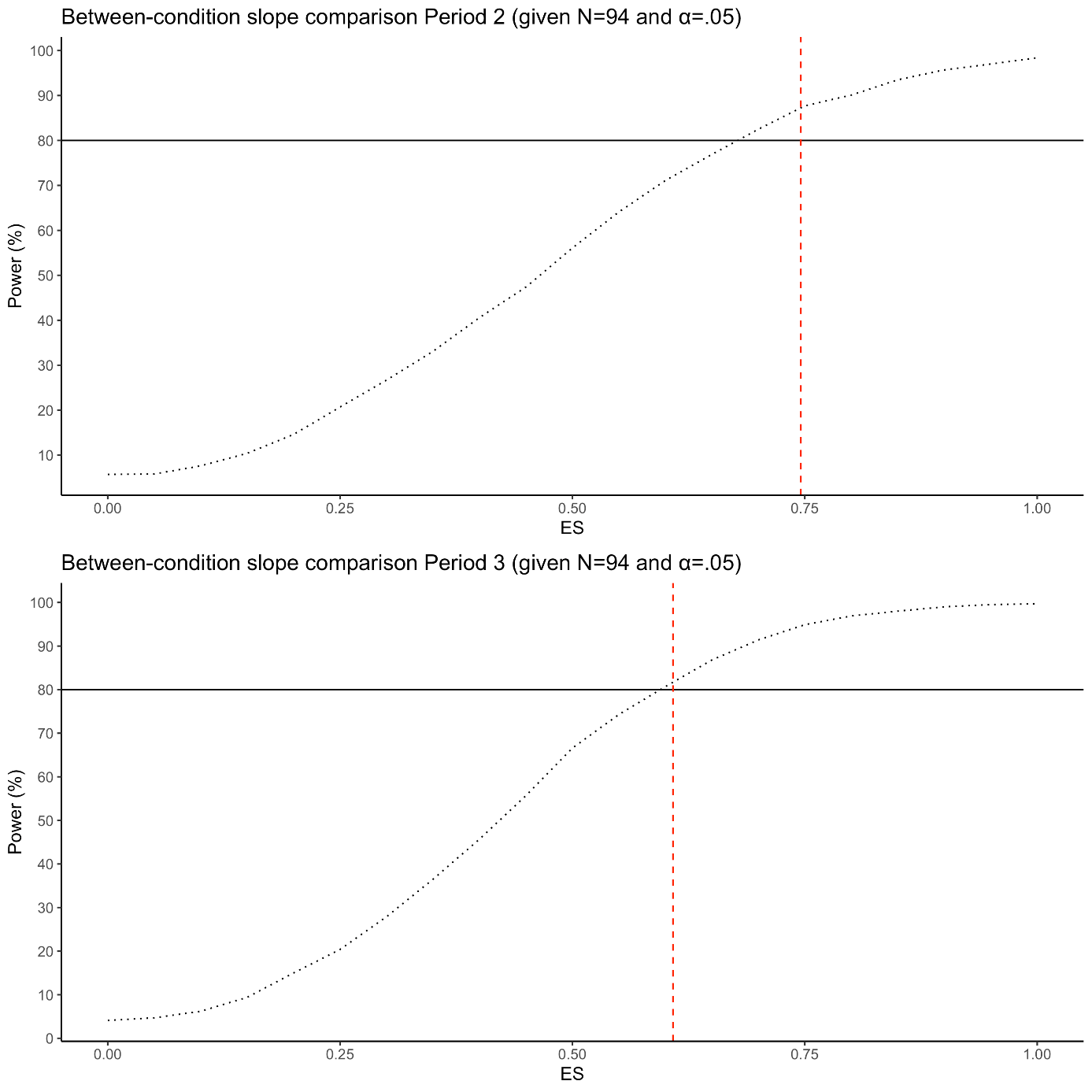


Note: Statistical power was computed for period x condition interactions assuming N = 94 and significance levels of α = 0.05. Remaining fixed effect estimates, residual variation and intercept variation were held constant as statistical power was estimated for a continuous effect size spectrum in 1000 iterations of Monte-Carlo simulation. Effect sizes for slope differences were defined as Cohen’s d as suggested by Westfall and colleagues (2018). Solid black lines represent critical power threshold of 80%, i.e. Type-II error probabilities of 20%,  black dotted lines represent results of Monte Carlo-based power simulation. The red dashed lines represent the observed effects within the sample
